# Facilitated Subcutaneous Immunoglobulin Treatment in Patients with Immunodeficiencies: the FIGARO Study

**DOI:** 10.1007/s10875-023-01470-2

**Published:** 2023-04-10

**Authors:** Michael Borte, Leif G. Hanitsch, Nizar Mahlaoui, Maria Fasshauer, Dörte Huscher, Matthaios Speletas, Maria Dimou, Marta Kamieniak, Corinna Hermann, David Pittrow, Cinzia Milito

**Affiliations:** 1 IDCL (ImmunoDeficiency Center Leipzig), Hospital for Children and Adolescents, St. Georg Hospital, Academic Teaching Hospital of the University of Leipzig, Leipzig, Germany; 2 Institute of Medical Immunology, Charité Universitätsmedizin Berlin, Berlin, Germany; 3 Pediatric Immunology-Hematology and Rheumatology Unit and French National Reference Center for Primary Immune Deficiencies (CEREDIH), Necker Children’s University Hospital, Assistance Publique-Hôpitaux de Paris (AP-HP), Paris, France; 4 Institute of Biometry and Clinical Epidemiology, Berlin Institute of Health, Charité Universitätsmedizin Berlin, Berlin, Germany; 5 Faculty of Medicine, Department of Immunology and Histocompatibility, Faculty of Medicine, School of Health Sciences, University of Thessaly, Larissa, Greece; 6 First Department of Propaedeutic Internal Medicine, General Hospital “LAIKO”, National & Kapodistrian University of Athens Medical School, Athens, Greece; 7 Takeda Development Center Americas, Inc, Cambridge, MA USA; 8 Baxalta Innovations GmbH, a Takeda Company, Vienna, Austria; 9 Institute for Clinical Pharmacology, Medical Faculty, Technical University of Dresden, Dresden, Germany; 10 Innovation Center Real World Evidence, GWT-TUD GmbH, Dresden, Germany; 11 Department of Molecular Medicine, Sapienza University of Rome, Rome, Italy

**Keywords:** Facilitated subcutaneous immunoglobulin, Immunoglobulin replacement therapy, Primary immunodeficiency disease, Secondary immunodeficiency disease, Utilization pattern

## Abstract

**Purpose:**

The FIGARO study aims to provide insights on real-world utilization and tolerability of facilitated subcutaneous immunoglobulin (fSCIG) for primary immunodeficiency disease (PID) or secondary immunodeficiency disease (SID).

**Methods:**

This prospective, multicenter, observational study, evaluated medical records, charts, and diaries of patients who had received at least 1 fSCIG infusion for PID or SID. Data were analyzed by cohort (PID, SID) and age groups (pediatric [< 18 years], adult [18–64 years], older adult [≥ 65 years]). Patients were followed up to 36 months.

**Results:**

The study enrolled 156 patients: 15 pediatric, 120 adult, 21 older-adult. Twelve-month follow-up data were available for 128 patients. fSCIG was mainly prescribed for PID among patients aged < 65 years and for SID among older adults. At inclusion, 75.6% received their fSCIG infusion at home, and 78.7% self-administered. Adults were more likely to receive their initial infusion at home and self-administer (81.7% and 86.6%, respectively) than pediatric patients (53.3% each) and older adults (57.1% and 52.4%, respectively). At 12 months, the proportion of patients infusing at home and self-administering increased to 85.8% and 88.2%. Regardless of age, most patients self-administered the full fSCIG dose at home every 3–4 weeks and required a single infusion site. The tolerability profile was consistent with previous pivotal trials. Acute severe bacterial infections occurred in 0%–9.1% of patients during follow-up visits (full cohort).

**Conclusions:**

FIGARO confirms the feasibility, tolerability, and good infection control of fSCIG in PID and SID patients across the age spectrum in both the home-setting and medical facility.

**Trial registration number:**

ClinicalTrials.gov NCT03054181

**Supplementary Information:**

The online version contains supplementary material available at 10.1007/s10875-023-01470-2.

## Introduction

Primary immunodeficiency (PID) and secondary immunodeficiency (SID) diseases are groups of heterogenous disorders characterized by failure or absence of components of the immune system, leading to, among other manifestations, chronic and/or recurrent infections [1]. Immunoglobulin replacement therapy (IGRT) is the standard of care for patients with PID with impaired antibody production [2], and is recommended by treatment guidelines for SID among patients with recurrent infections despite prophylactic oral antibiotic therapy [3]. IGRT can be administered intravenously (IVIG) or subcutaneously (SCIG). SCIG has similar efficacy to IVIG but does not require venous access and is associated with fewer systemic adverse drug reactions (ADRs). SCIG can be self-administered at home. Conventional SCIG (cSCIG) requires frequent infusions (daily, weekly, or biweekly) and volume limitations often necessitate administration into multiple sites [2, 4].

Facilitated subcutaneous immunoglobulin (fSCIG) is a dual-vial unit of recombinant human hyaluronidase (rHuPH20) and 10% human normal immunoglobulin G (IgG) [5, 6]. rHuPH20 depolymerizes hyaluronan, increasing subcutaneous tissue permeability and allowing infusion of larger volumes of IgG compared to cSCIG. As a result, fSCIG can be self-administered at home every 3 to 4 weeks using a single infusion site [5, 6].

Although data on efficacy and safety of fSCIG in SID are limited [7], a pivotal phase 3 study of patients aged 4–78 years with PID (NCT00814320) demonstrated that fSCIG was effective and bioequivalent to IVIG at the same administration intervals, with fewer systemic reactions [8]. Several retrospective real-world studies have confirmed the feasibility and tolerability of fSCIG in patients with PID or SID in routine clinical practice, including in children and older adults [9–12]; however, a comprehensive prospective analysis of the use of fSCIG among patients with PID or SID in the real-world has not been published. To provide in-depth insights on the real-world utilization, safety, and tolerability of fSCIG among patients with PID or SID across the age spectrum, we conducted the large-scale Facilitated Immunoglobulin Administration Registry And Outcomes (FIGARO) study across Europe under the auspices of the European Society for Immunodeficiencies.

## Materials and Methods

### Patients and Study Design

FIGARO was a prospective, observational, phase 4 study conducted in 14 centers in 6 European countries: Czech Republic, Germany, Greece, Italy, Poland, and Spain. Study initiation was December 2016; database closure was August 2021. Duration of follow-up was dependent on when the patient was included during the study period. Patients were followed for up to 36 months. Patients were eligible to enroll if they had received at least one fSCIG infusion for PID or SID or were due to receive one and met the following criteria: indication for IGRT, available for long-term follow-up, and provided informed consent. Note that due to center selection, there was likely a bias towards enrolment of a higher number of patients with PID versus those with SID.

The primary objective was to assess drug utilization patterns including dose, treatment interval, infusion volume, infusion sites, infusion rate, needle size, site of care, type of pump, and caregiver support. Concomitant medications per Anatomic Therapeutic Chemical (ATC) code and disease states per Medical Dictionary for Regulatory Activities (MedDRA) code, serum trough levels, premedication prior to fSCIG infusion, infections, adverse drug reactions (ADRs), training sessions, and nurse visits at home were also assessed.

### Assessments

Data were collected from patient charts, diaries, and patient interviews and entered into an online form at regular intervals. Centers were required to perform complete data entries one to four times per year. Data were collected on parameters, including patient demographics, clinical characteristics, and IGRT treatment history. Physician interpretation of serum IgG trough levels was evaluated using a rating scale of “too low,” “satisfactory,” “optimal,” or “too high.” Infections, including acute severe bacterial infection (ASBI) events and ADRs (categorized as local or systemic) were also evaluated. Healthcare utilization was assessed by recording number of training sessions, nurse visits, patient visits, hospitalizations and rehabilitations, and sickness days.

### Statistical Methods

The sample size was determined by feasibility aspects. Analyses were performed for the overall population, PID and SID cohorts, and for subgroups stratified by age (< 18 years [pediatric], 18–64 years [adults], ≥ 65 years [older adults]). Subgroup analyses are reported to 12 months due to small patient numbers in some subgroups at later follow-ups. Follow-up visits were allocated to 6-month intervals. To allocate the 591 follow-up visits to the 6-month intervals of interest, in some cases up to three follow-up visits had to be combined for one interval. Available information for each interval was aggregated as follows: time lag to baseline – last documentation date; unknown information – best status available; interruptions, problems, health sources use, infections – ever mentioned/used; above related durations, counts – sum over all documentations (usually “since last visit”); fSCIG discontinuation – the first in sequence of reasons listed; fSCIG adherence – best status; IgG trough level – mean value; IgG trough level interpretation – the last in sequence of categorizations listed. All general information about current fSCIG use was calculated based on weighted documentations. Descriptive analyses were performed with continuous numeric variables expressed as number of evaluable values, mean and standard deviation (SD), or median and range. Categorical variables were described as absolute and relative frequency counts. No specific statistical hypothesis testing was performed. No imputations for missing values were made. A sensitivity analysis that excluded patients in the ramp-up (titration) phase at the inclusion visit evaluated dose, infusion volume, infusion rate, and treatment interval at the inclusion visit and during follow-up.

## Results

### Patients

**Fig. 1:**
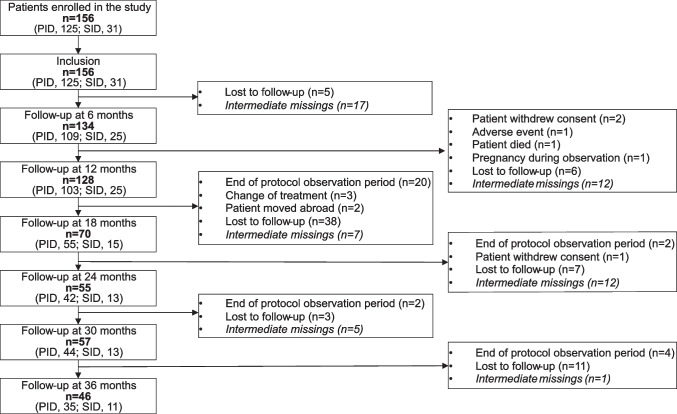
Patient disposition. “Intermediate missing” indicates that visits between available visits are missing. (e.g., a patient has no 12-month visit, but data was available for the 18-month visit.). PID, primary immunodeficiency disease; SID, secondary immunodeficiency disease

A total of 156 patients were included, 125 patients in the PID cohort and 31 patients in the SID cohort. Patient demographics and clinical characteristics at inclusion are summarized in Table 1, and patient disposition over the study duration is summarized in Fig. 1. The mean observation duration was 20.3 ± 12.0 months (median 14.5 months), with 128, 55, and 46 patients providing data at 12, 24, and 36 months of follow-up, respectively**.** Patients’ mean age was 42.4 ± 19.1 years: 15 patients were pediatric (< 18 years; median [min, max]: 9.0 [1.0, 17.0] years), 120 patients were adults (18–64 years; 41.0 [18.0, 64.0] years), and 21 patients were older adults (≥ 65 years; 72.0 [65.0, 88.0] years). PID was the predominant indication for IGRT among patients aged < 65 years (pediatric: 93.3%; adults: 87.5%), and SID among older adults (71.4%).

**Table 1:** Demographic and clinical characteristics at inclusion by age subgroup

Parameter	Age group			
	< 18 years (*n* = 15)	18–64 years (*n* = 120)	≥ 65 years (*n* = 21)	Total (*n* = 156)
Demographics				
Age, mean (SD) (years)	9.9 (4.8)	41.2 (12.9)	72.7 (6.2)	42.4 (19.1)
Male, *n* (%)	6 (40.0)	62 (51.7)	14 (66.7)	82 (52.6)
Caucasian/White, *n* (%)	14 (93.3)	115 (99.1)	20 (95.2)	149 (98.0)
Clinical characteristics				
BMI, mean (SD) (kg/m^2^)	19.6 (5.7)	24.8 (4.6)	26.2 (4.1)	24.5 (4.9)
Indication for IGRT, *n* (%)				
PID	14 (93.3)	105 (87.5)	6 (28.6)	125 (80.1)
CVID	6 (42.9)	84 (79.2)	5 (83.3)	95 (76.0)
X-linked agammaglobulinemia (Bruton disease)/ARA	1 (7.1)	9 (8.6)	0	10 (8.0)
Isolated IgG subclass deficiency	0	1 (1.0)	1 (16.7)	2 (1.6)
Specific antibody deficiency with normo- or hypogammaglobulinemia	3 (21.4)	1 (1.0)	0	4 (3.2)
CSR defects/Hyper-IgM syndrome	1 (7.1)	1 (1.0)	0	2 (1.6)
Unclassified antibody deficiency	0	4 (3.8)	0	4 (3.2)
Combined IgA/IgG subclass deficiency	0	2 (1.9)	0	2 (1.6)
Other	3 (21.4)	3 (2.9)	0	6 (4.8)
SID	1 (6.7)	15 (12.5)	15 (71.4)	31 (19.9)
CLL	0	8 (53.3)	12 (80.0)	20 (64.5)
Indolent lymphoma	0	1 (6.7)	3 (20.0)	4 (12.9)
Other SID^a^	1 (100.0)	6 (40.0)	0	7 (22.6)
Received chemotherapy, immunosuppressive therapy, or supportive therapy, n (%)	11 (73.3)	93 (77.5)	21 (100.0)	125 (80.1)
Concomitant supportive therapy, n (%)				
Antibiotics	4 (26.7)	22 (18.3)	4 (19.0)	30 (19.2)
Corticosteroids	1 (6.7)	8 (6.7)	1 (4.8)	10 (6.4)
Expectorants	0	1 (0.8)	1 (4.8)	2 (1.3)
Inhalation therapy	1 (6.7)	20 (16.7)	3 (14.3)	24 (15.4)
PJP prophylaxis	0	5 (4.2)	10 (47.6)	15 (9.6)
Virostatics	1 (6.7)	6 (5.0)	8 (38.1)	15 (9.6)
Other supportive therapy	3 (20.0)	22 (18.3)	7 (33.3)	32 (20.5)
IGRT history^b^				
Reason for discontinuation of previous IGRT,^c^ n (%)				
Patient request	9 (60.0)	74 (40.2)	6 (35.3)	89 (41.2)
Other	3 (20.0)	46 (25.0)	7 (41.2)	56 (25.9)
Tolerability	2 (13.3)	27 (14.7)	3 (17.6)	32 (14.8)
Administrative	1 (6.7)	20 (10.9)	0	21 (9.7)
Lack of efficacy	0	17 (9.2)	1 (5.9)	18 (8.3)

*ARA* autosomal-recessive agammaglobulinemia, *BMI* body mass index, *CLL* chronic lymphatic leukemia, *CSR* Class-Switch Recombination, *CVID* common variable immunodeficiency disease, *fSCIG* facilitated subcutaneous immunoglobulin, *Ig* immunoglobulin, *IGRT* immunoglobulin replacement therapy, *IV* intravenous, *PID* primary immunodeficiency diseases, *PJP* pneumocystis jirovecii pneumonia, *SC* subcutaneous, *SD* standard deviation, *SID* secondary immunodeficiency diseases

^a^Other SID includes B-NHL; diffuse large B-cell lymphoma; Hodgkin’s disease; Hodgkin’s lymphoma after autologous transplantation; Lu-Tx, rituximab therapy; rituximab; and secondary immunodeficiency due to immunosuppressive therapy of rheumatism (n = 1 for all). ^b^Multiple responses possible. For therapies other than fSCIG. ^c^Data refer to the number of IV and SC treatments named rather than the number of patients

Most patients (89.7%) had at least one concomitant disease,  if any (mean of 3.8 ± 2.3) (Supplemental Table 1). Chemotherapy and immunosuppressive therapies were more frequent among patients with SID, with any chemotherapy, immunotherapy, or supportive therapy being reported in 29 patients in the SID cohort (93.5%) and 96 patients in the PID cohort (76.8%). In the full cohort, the highest proportion of patients receiving these therapies were those aged ≥ 65 years (Table 1). During follow-up, concomitant medication use was stable. Patient request (41.2%) was the most common reason for prior IGRT discontinuations (Table 1).

### fSCIG Dose and Administration

**Fig. 2:**
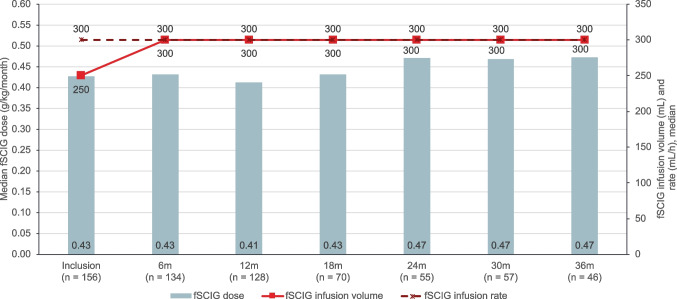
fSCIG dose, infusion volume, and rate in the total population over 36 months of follow-up. *n* values represent number of patients at each visit; *n* values for each parameter may differ slightly due to missing data for that individual parameter. fSCIG, facilitated subcutaneous immunoglobulin

**Fig. 3:**
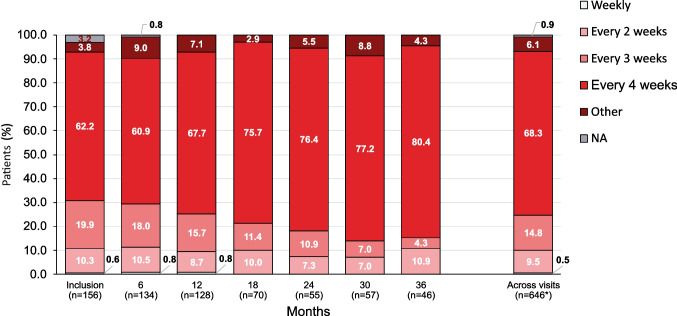
fSCIG infusion interval in the total population over 36 months of follow-up. n values represent number of patients at each visit; n values for each parameter may differ slightly due to missing data for that individual parameter. *Across visits, information is available for *n *= 644 visits. fSCIG, facilitated subcutaneous immunoglobulin; NA, not applicable as the patients received only one fSCIG infusion to date

**Fig. 4:**
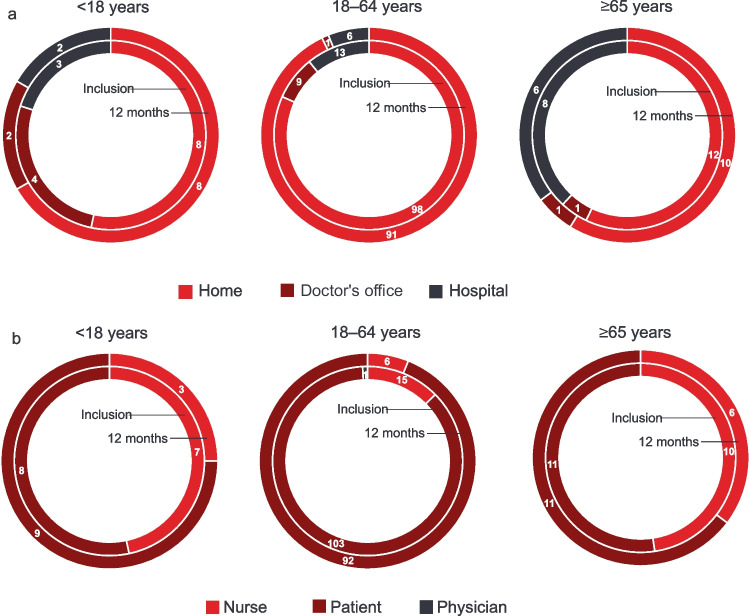
fSCIG administration by age subgroup at inclusion and 12 months. **a**) Administration setting; **b**) Infusion administrator. Note: Among the 11 patients in the ramp-up phase at inclusion, 7 patients (63.6%) received their most recent fSCIG infusion at the doctor’s office and 4 patients (36.4%) at the hospital. Most patients (81.8% [9/11]) received their most recent infusion from a nurse, 1 patient (9.1%) from a physician, and 1 patient (9.1%) self-administered. Inner circle denotes at inclusion; outer circle denotes at 12 months. Data labels are number of patients. fSCIG, facilitated subcutaneous immunoglobulin

For naïve patients and for patients switching from SCIG therapy, it is recommended that the treatment intervals for the first infusions are gradually prolonged from a 1-week dose to up to a 3- or 4-week dose. At inclusion, 11 patients (7.1%) were in the ramp-up (titration) phase (PID: 10 [8.1%]; SID: 1 [3.3%]). The median fSCIG dose by body weight at inclusion was 0.43 g/kg per month (range: 0.11–0.83). Older adults tended to infuse at a slightly lower dose by body weight than patients < 65 years (Table 2). This in part may have been due to the lower median monthly dose used for the SID patients compared to the PID patients (0.40 vs 0.43 g/kg). During follow-up, the median fSCIG dose remained relatively stable throughout all visits. At 36 months, the median monthly dose increased to a maximum value of 0.51 g/kg per month in the PID cohort and decreased to 0.37 g/kg in the SID cohort (Fig. 2). Patients predominantly infused fSCIG every 3–4 weeks (82.1% at inclusion), with more patients moving to every 4-week infusion intervals over time (Fig. 3). At inclusion, 75.6% of patients received their fSCIG infusion at home and 78.7% self-administered, increasing to 85.8% and 88.2% by 12 months, respectively, and 93.5% and 95.7% by 36 months, respectively. At the most recent visit, patients in the PID cohort were more likely to self-administer fSCIG (83.9% vs 58.1%) at home (79.2% vs 61.3%) than patients in the SID cohort. Adults were more likely to have received their most recent infusion at home and self-administer than pediatric patients and older adults**.** The proportion of pediatric patients self-administering at home tended to increase over follow-up (Fig. 4). Premedication prior to fSCIG infusion was rarely required (2.6% at inclusion [PID: 2.4%; SID: 3.2%]; 2.2% of all follow-up visits combined [PID: 2.1%; SID: 3.0%]). Adherence with fSCIG was high, with almost 95% of patients with available data infusing on (69.7%) or within ± 1–3 days (25.0%) of the scheduled date at inclusion.

**Table 2:** fSCIG dosing and infusion parameters at inclusion and 12 months by age subgroups

Parameter, median (range)	< 18 years		18–64 years		≥ 65 years		Total	
	Inclusion	12 months	Inclusion	12 months	Inclusion	12 months	Inclusion	12 months
	(*n *= 15)	(*n* = 12)	(*n* = 120)	(*n* = 99)	(*n* = 21)	(*n* = 17)	(*n* = 156)	(*n* = 128)
Total fSCIG dose at the most recent infusion, g	10.0 (2.5–30.0)	13.8 (5.0–40.0)	25.0 (10.0–60.0)	30.0 (10.0–60.0)	30.0 (20.0–50.0)	30.0 (15.0–50.0)	25.0 (2.5–60.0)	30.0 (5.0–60.0)
fSCIG total monthly dose, g	15.0 (10.0–30.0)	15.0 (5.0–40.0)	30.0 (10.0–75.0)	30.0 (10.0–60.0)	30.0 (20.0–50.0)	30.0 (1.0–50.0)	30.0 (10.0–75.0)	30.0 (1.0–60.0)
fSCIG dose, g/kg/month	0.476 (0.106–0.833)	0.500 (0.341–0.645)	0.423 (0.169–0.816)	0.425 (0.116–0.857)	0.410 (0.250–0.545)	0.349 (0.012–0.526)	0.427 (0.106–0.833)	0.412 (0.012–0.857)
Total fSCIG infusion volume^a^, mL	100 (25–300)	138 (50–400)	300 (10–600)	300 (25–600)	300 (40–350)	300 (30–400)	250 (10–600)	300 (25–600)
fSCIG maximum infusion rate, mL/h	100 (10–300)	160 (50–300)	300 (60–300)	300 (60–320)	300 (240–300)	275 (240–300)	300 (10–300)	300 (50–320)
Number of infusion sites	1.0 (1.0–2.0)	1.0 (1.0–2.0)	1.0 (1.0–2.0)	1.0 (1.0–2.0)	1.0 (1.0–2.0)	1.0 (1.0–2.0)	1.0 (1.0–2.0)	1.0 (1.0–2.0)
Infusion location, *n* (%)								
Upper abdomen	5 (33.3)	3 (25.0)	63 (52.5)	54 (54.0)	4 (19.0)	6 (32.4)	72 (46.2)	62 (48.4)
Lower abdomen	5 (33.3)	0 (0)	35 (29.2)	15 (15.2)	11 (52.4)	3 (17.6)	51 (32.7)	18 (14.1)
Thigh	4 (26.7)	4 (33.3)	3 (2.5)	5 (4.5)	0	0	7 (4.5)	9 (6.6)
Other	1 (6.7)	5 (41.7)	0	0	0	0	1 (0.6)	5 (3.9)
Unknown	0	0	19 (15.8)	26 (26.3)	6 (28.6)	9 (50.0)	25 (16.0)	35 (27.0)
Infusion interval, n (%)								
Weekly	0	0	1 (0.8)	1 (1.0)	0	0	1 (0.6)	1 (0.8)
Every 2 weeks	0	0	16 (13.3)	11 (11.2)	0	0	16 (10.3)	11 (8.7)
Every 3 weeks	3 (20.0)	0	27 (22.5)	19 (19.4)	1 (4.8)	1 (5.9)	31 (19.9)	20 (15.7)
Every 4 weeks	12 (80.0)	12 (100.0)	68 (56.7)	61 (62.2)	17 (81.0)	13 (76.5)	97 (62.2)	86 (67.7)
Other^b^	0	0	4 (3.3)	6 (6.1)	2 (9.5)	3 (17.6)	6 (3.8)	9 (7.1)
NA^c^	0	0	4 (3.3)	0	1 (4.8)	0	5 (3.2)	0
IgG serum trough level, median (IQR) g/L	7.0 (6.6–9.4)	8.1 (7.1–8.8)	8.4 (7.1–9.5)	8.2 (7.3–9.3)	6.9 (4.8–9.0)	7.0 (4.6–8.5)	8.1 (6.8–9.5)	8.1 (7.2–9.2)

*fSCIG* facilitated subcutaneous immunoglobulin, *Ig* immunoglobulin, *IQR* interquartile range

^a^Total infusion volume over all sites per patient. ^b^Other: comprises intervals up to every 35 weeks. ^c^Not applicable, as the patients received only one fSCIG infusion to date

Median IgG serum trough levels remained relatively constant across all age subgroups, ranging from 6.9 to 10.4 g/L. At inclusion, the mean IgG serum trough level in the full cohort was 8.3 ± 2.5 g/L. Trough levels were higher in the PID cohort (mean 8.7 ± 2.4) than in the SID cohort (6.0 ± 1.8) which may be related to the lower dose/kg seen in the SID cohort at inclusion. During follow-up, serum IgG trough levels tended to increase (maximum mean value 9.1 ± 2.1 g/L). The effect was driven by the increase in levels in the SID group (maximum mean value 8.7 ± 3.3 g/L).

### fSCIG Infusion Parameters

The median fSCIG infusion volume per patient was 250 mL (range: 10 mL–600 mL) at inclusion, increasing to 300 mL at 6 months after which it remained constant across visits (Fig. 2). Median maximal infusion rate remained constant at 300 mL/h over the course of the study with no differences in PID and SID cohorts from 18 to 36 months. As anticipated, compared with adults, pediatric patients initiated fSCIG at a lower median infusion volume and maximum infusion rate (100 mL and 100 mL/h, respectively), which at 12 months increased to 138 mL and 160 mL/h (Table 2).

Over the course of the study, most patients used a single infusion site (most commonly in the upper or lower abdomen) and a 24-gauge needle with a length of 9–12 mm**.** Across all visits, the majority of all fSCIG infusions were administered using a pump (97.9% overall). Technical problems were rare (*n* = 20 infusions; 3.2% of all infusions), and over the course of the study, the full planned fSCIG dose was administered for 99.1% of infusions.

### Sensitivity Analysis Excluding Patients in the Ramp-up Phase at the Inclusion Visit

Among the 11 patients in the ramp-up phase at inclusion, 7 patients (63.6%) received their most recent fSCIG infusion at the doctor’s office and 4 patients (36.4%) at the hospital; there were no patients ≥ 65 years of age in the ramp-up phase at inclusion. The most recent ramp-up infusion was administered by a nurse in 81.8% (9/11) of patients, 1 patient (9.1%) received the most recent infusion from a physician, and 1 patient (9.1%) self-administered.

In the sensitivity analysis that excluded these 11 patients, the median fSCIG dose by body weight was 0.42 g/kg per month (range: 0.11–0.83) at inclusion, with pediatric patients infusing at higher dose by body weight than adult patients (Supplemental Table 2). The median fSCIG dose by body weight remained relatively stable over 36 months of follow-up (Supplemental Fig. S1). Most patients (83.4%) infused their most recent fSCIG infusion every 3–4 weeks at inclusion, and the proportion of patients infusing every 4 weeks tended to increase over time (Supplemental Fig. S2).

At inclusion, 81.4% of patients received their fSCIG infusion at home and 84.0% self-administered, increasing to 85.0% and 88.3% by 12 months, respectively, and 93.0% and 95.3% by 36 months, respectively (Supplemental Fig. S3). Adults 18–64 years of age were more likely to have received their most recent infusion at home and to self-administer than pediatric patients and older adults at inclusion, and the proportion of pediatric patients who self-administered their infusions increased during follow-up (Supplemental Fig. S3).

The median fSCIG infusion volume per patient was 300 mL (range: 10 mL–600 mL) at inclusion and remained constant across all visits during follow-up. Median maximal infusion rate remained constant at 300 mL/h at all study visits (Supplemental Fig. S1). Pediatric patients initiated fSCIG at a lower median infusion volume and maximum infusion rate (137.5 mL and 126.5 mL/h, respectively) compared with adults (300 mL and 300 mL/h); at 12 months, the median infusion volume and infusion rate for pediatric patients increased to 175 mL and 171.0 mL/h, respectively (Supplemental Table 2).

### Safety

**Fig. 5:**
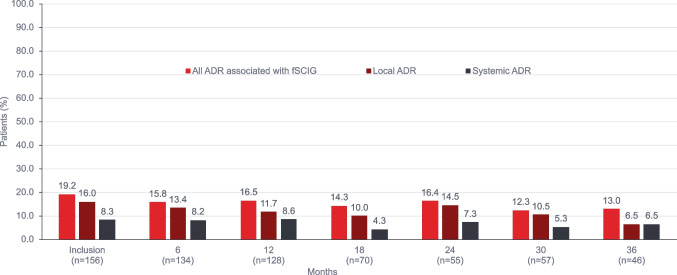
Adverse reactions in the total population over 36 months of follow-up. Multiple reactions possible. ^a^Local (infusion site) includes infusion site erythema, inflammation, infusion site itching. ^b^Systemic (generalized or non-infusion site) includes acute diarrhea, aseptic meningitis, chills, dizziness, drowsiness, fatigue, fever, fever chills, flu-like symptoms, headache, hypertension, itching, malaise, vasovagal reaction, weakness. ADR, adverse drug reactions; fSCIG, facilitated subcutaneous immunoglobulin

fSCIG-associated ADRs were reported by 30 patients (19.2%) at inclusion; 25 patients (16.0%) had local ADRs (infusion site inflammation in all cases), and 13 patients (8.3%) had systemic ADRs (most commonly flu-like symptoms)**.** Most patients with SID did not report ADRs associated with fSCIG infusion; one patient reported a local ADR (infusion site inflammation), and one patient had a systemic ADR (severe headache) at the inclusion visit. At 36 months, ADRs associated with fSCIG infusions were reported in 6 patients (17.1%) in the PID cohort compared to no patients in the SID cohort. fSCIG-associated ADRs over 36 months are summarized in Fig. 5. ADRs by age group and by administration setting at inclusion and 12 months are summarized in Supplemental Tables 3 and 4.

### Infections

ASBI events occurring in the study are listed in Supplemental Table 5. In the full cohort, 18/156 patients (11.5%; PID: 8/125 [6.4%] patients, SID: 10/31 [32.3%] patients; age-wise distribution: < 18 years 3/15 patients [20.0%], 18–64 years 10/120 patients [8.3%], ≥ 65 years 5/21 patients [23.8%]) reported one or more ASBI events in the 12 months prior to inclusion. Among the 8 patients in the PID cohort reporting ASBI events, 4 patients had < 12 months of IGRT and 4 patients had ≥ 12 months of IGRT prior to inclusion to FIGARO. Among the 10 patients in the SID cohort reporting ASBI events, 8 patients had < 12 months of IGRT and 2 patients had ≥ 12 months of IGRT prior to inclusion to FIGARO. There were a total of 26 ASBI events at inclusion (PID: 12 events, SID: 14 events). Pneumonia was the most frequently reported ASBI event before entering the FIGARO study (15/26 events [57.7%]; PID: 10/12 [83.3%] events, SID: 5/14 [35.7%] events).

At 6, 12, 18, 24, and 30 months, ASBI events were reported in 6/134 (4.5%), 4/128 (3.1%), 5/70 (7.1%), 5/55 (9.1%), and 3/57 (5.3%) patients, respectively. No ASBI events were reported at 36 months. All ASBI events occurred in the PID cohort. There were 14 patients who reported ASBI events at one study visit, 3 patients who reported ASBI events at two study visits, and 1 patient who reported ASBI events at three study visits at follow-up, with most patients reporting one or two ASBI events at each visit.

At follow-up, hospitalization was required for 1/7 events (14.3%; lung abscess: 29 days in hospital) at 6 months (hospitalization data unknown for 1 event), 1/5 events (20.0%; pneumonia: 6 days in hospital) at 12 months (hospitalization data unknown for 1 event), 2/5 events (40.0%; acute diarrhea: 4 days in hospital; pneumonia: 11 days in hospital) at 18 months, and for 1/3 events (33.3%; urinary tract infection: 3 days in hospital) at 30 months.

In the 12 months prior to inclusion, other bacterial infection events were reported in 83/156 patients (53.2%; PID: 72/125 [57.6%] patients; SID: 11/31 [35.5%] patients; age-wise distribution: < 18 years 8/15 patients [53.3%], 18–64 years 68/120 patients [56.7%], ≥ 65 years 7/21 patients [33.3%]). There were a total of 159 other bacterial infection events (PID: 132 events; SID: 27 events). The most frequently reported infections were sinusitis (39/159 events [24.5%]; PID: 32/132 [24.2%] events, SID: 7/27 [25.9%] events), bronchitis (23/159 events [14.5%]; PID: 23/132 [17.4%] events), and pharyngitis (9/159 events [5.7%]; PID: 8/132 [6.1%] events, SID: 1/27 [3.7%] event).

At 6, 12, 18, 24, 30, and 36 months, other bacterial infection events were reported in 34/134 (25.4%), 36/128 (28.1%), 13/70 (18.6%), 16/55 (29.1%), 11/57 (19.3%), and 9/46 (19.6%) patients, respectively. Overall, the rate of other bacterial infections at 36 months was higher in the PID cohort (22.9%) than in the SID cohort (9.1%).

At follow-up, hospitalization was required in 4/43 other bacterial infection events (9.3%; PID: inguinal abscess: 1 day in hospital; gastroenteritis: 4 days in hospital; COVID-19: 7 days in hospital; and upper respiratory tract infection: 2 days in hospital) at 6 months (hospitalization data unknown for 2 events), 2/45 events (4.4%; PID: bacterial infection: 3 days in hospital; SID: appendicitis: 10 days in hospital) at 12 months (hospitalization data unknown for 1 event), 1/15 events (6.7%; PID: diarrhea: 19 days in hospital) at 18 months (hospitalization data unknown for 3 events), and 1/22 events (4.5%; cholangitis: 7 days in hospital) at 24 months (hospitalization data unknown for 1 event). No hospitalizations were documented at 30 and 36 months for other bacterial infection events.

### Training and Administration Health Resource Utilization

At inclusion, patients received a mean of 2.0 ± 2.3 (range 0–12) nurse training sessions for the correct administration of fSCIG. Training sessions were higher in the PID cohort (2.0 ± 2.3, range 0–12) than in the SID cohort (mean 1.8 ± 2.1, range 0–5). The mean number of nurse visits to the home to administer fSCIG was 0.4 ± 1.1 (range 0–4). Home administration visits were lower in the PID cohort (0.3 ± 0.9, range 0–4) than in the SID cohort 1.0 ± 1.5 (range 0–4). Similar data were observed across all age subgroups. Nurse home administration of fSCIG was low across PID and SID cohorts over the follow-up, and training sessions were not needed after 12 months except for one training session in a pediatric patient.

## Discussion

The FIGARO study provides a comprehensive examination of the safety, tolerability, and utilization of fSCIG in the real-world setting. Results presented here demonstrate that fSCIG allowed for flexibility in dosing and administration setting. Most patients in FIGARO self-administered fSCIG at home via infusion pump, with the majority infusing every 3–4 weeks into a single site. The majority of patients were able to infuse fSCIG without any technical problems with the full planned dose being administered (99.1%). Ease of administration is further supported by the few training sessions and nurse visits that were required for fSCIG administration across cohorts. Previous studies have demonstrated that patients with PID and their caregivers prefer less frequent infusions, shorter administration durations, fewer needlesticks, the ability to administer treatments at home, and the option to self-administer [13]. Furthermore, patients or their caregivers expressed a preference for fSCIG to alternative treatment modalities [14]. Findings from this real-world study highlight the potential benefits of individualizing treatment with fSCIG for patients with PID and SID by accounting for their treatment needs and preferences.

Although patients across subgroups predominantly received fSCIG treatment every 3–4 weeks and used a single infusion site, some differences between groups were identified. At inclusion, adults were more likely to receive fSCIG infusion at home and self-administer than pediatric patients and older adults. At the 12-month follow-up visit, infusion volume and maximal infusion rates were consistent with inclusion visit values for adults and older adults. These values tended to increase in pediatric patients (to 138 mL and 160 mL/h, respectively); this suggests good tolerance of the higher volume and a preference for shorter infusion duration. At 12 months, the most frequent infusion location was the upper abdomen overall; however, pediatric patients most frequently used an infusion location other than the upper abdomen, lower abdomen, or thigh. Home treatment and self-treatment with fSCIG were possible in most older adults (including elderly), as these patients administered the infusion at home with good tolerability using one infusion site. Minor differences were observed between PID and SID cohorts. The proportion of patients administrating fSCIG at home and self-administering was higher in the PID cohort than the SID cohort at baseline, but these differences disappeared during follow-up. Patients with SID were more likely to receive their infusions on the scheduled date. The number of nurse training sessions was initially higher in PID than the SID cohort, but training visits were no longer needed in either patient group after 12 months; while the number of nurse home visits to administer fSCIG was lower in the PID cohort than in the SID cohort. Regardless of these differences, dose, infusion volume, and rate remained constant over time, as did IgG serum trough levels across subgroups.

A sensitivity analysis was conducted to evaluate the impact of the 11 patients who were in the ramp-up phase at the inclusion visit on median dose, infusion volume, infusion rate, treatment intervals, administration site, and administrator throughout the study. These patients were more likely to be receiving their infusion in a hospital or doctor’s office administered by a healthcare professional at inclusion compared with the overall population. Despite these differences, findings from the sensitivity analysis that excluded these patients were generally similar to results from the primary analysis during the follow-up period.

The efficacy of fSCIG in infection control in patients with PID has been demonstrated in clinical trials with adult and pediatric populations [14, 15]. The infection rate for the former was 2.97 infections per patient-year, and for the latter was 3.02 infections per patient-year. Real-world data in patients with SID show fSCIG administration to be effective in reducing infections, with 24.4% of patients experiencing ≥ 1 grade 2 infection episodes [7]. The results of the FIGARO study are consistent with previous findings demonstrating good infection control with 4.5% of patients reporting ASBI at 6 months, 3.1% at 12 months, 7.1% at 18 months, 9.1% at 24 months, and 5.3% at 30 months (all in the PID cohort). 22.9% of  the PID cohort and 9.1% of the SID cohort experienced other bacterial infections at 36 months.

The positive experience of the patients included in this study may have important implications on quality of life and independence. Overall, fSCIG was well tolerated and side effects were usually minor. After 36 months of follow-up, fSCIG-associated local ADRs were reported by 6.5% of patients; all local ADRs were related to infusion site inflammation. Systemic ADRs were reported in 7.4% of all visits, with flu-like symptoms, fatigue, fever, and headache most commonly reported. No serious ADRs occurred. ADRs led to discontinuation of fSCIG in only one case. Events of ASBI during follow-up occurred in 18 (11.5%) patients; all were in the PID cohort and most reported ASBI events at only one or two study visits.

Premedication was rarely used (across all visits, 2.3%) supporting infusion tolerance. The ADR profile was consistent with that observed in previous pivotal trials [8, 14–16], and tolerability was confirmed across the age spectrum and in both the home and medical facility settings. The outcomes of the FIGARO study provide insights into the real-life, clinical utilization of fSCIG in patients with PID and SID from various countries, which aligns with previously reported outcomes. A retrospective multicenter analysis of medical records of patients aged ≥ 65 years with PID or SID requiring IGRT who received at least one infusion of fSCIG, SENEQA (Retrospective Data Collection of Elderly Patients Treated With HyQvia), found the majority of patients with PID *n* = 10 or SID *n* = 6 self-administered fSCIG (200 mL–350 mL) at home every 3–4 weeks using a single infusion site by infusion pump at rates up to 300 mL/h [10]. Another retrospective review of medical records of pediatric patients (aged < 18 years) with PID or SID at three centers in Germany receiving fSCIG, RAHPP (Retrospective chart Analysis of HyQvia usage in Pediatric Patients with PID or SID), found most (90%) patients received their first fSCIG infusion at a medical facility; by 6 months, all fSCIG infusions were administered at home by the patient/caregiver, the majority infusing every 3–4 weeks into a single site. No serious ADRs occurred [9]. Finally, a subset of patients from the SIGNS registry receiving fSCIG [17] confirmed the preparation was well tolerated and treatment satisfaction was high.

Taken together, data from the FIGARO study and previous reports imply fSCIG offers the ability to customize the treatment experience based on the patients’ needs and preferences and may allow patients to manage their treatment with fewer disruptions to their daily lives. With less frequent infusions and providing patients and healthcare providers the option to choose the site of care, including self-administration at home [14, 15], fSCIG may reduce treatment burden. fSCIG may increase patient convenience, as it allows for fewer administration sites and needles than conventional SCIG [14, 15, 18, 19]. These options could alleviate logistical and emotional burdens associated with receiving treatment in the hospital or doctor’s office for patients with PID and SID [19]. Specifically, having fewer interruptions to school, work, or recreational activities may promote feelings of independence and well-being [19, 20].

There are several limitations to consider in interpreting the study outcomes. This was a prospective, observational data collection with no control arm. Selection bias must be considered with respect to physicians and physician selection of patients deemed to be appropriate for subcutaneous treatment. In Europe, fSCIG is indicated for both PID and SID in patients of all ages; while in the United States, fSCIG is currently indicated only for use in adults with PID. FIGARO results may not be generalizable to other countries and regions with different licensed indications. As anticipated, the majority of patients with SID were in the older adult category, consistent with diagnosis of hematological malignancy; it is therefore difficult to distinguish whether any of the differences in utilization patterns in this group were attributable to the underlying indication for IGRT or to the age group. Additionally, the predominance of PID in the study does not align with the general population and is likely due to participant selection at the individual sites.

## Conclusions

FIGARO is the largest prospective, observational study of fSCIG to date, and confirms the feasibility and tolerability of fSCIG utilization in a broad range of patients with PID or SID across the age spectrum in the real-world setting. fSCIG provided patients with flexibility in dosing and administration at home or in a medical facility, according to patients’ underlying conditions and preferences, thereby allowing individualized treatment options. Regardless of age, most patients used a single infusion site and self-administered the full fSCIG dose at home every 3–4 weeks.

## Supplementary Information

Below is the link to the electronic supplementary material.

Supplemental Fig. S1 fSCIG dose, infusion volume, and rate over 36 months of follow-up, excluding patients in the ramp-up phase at the inclusion visit. n values represent number of patients at each visit; n values for each parameter may differ slightly due to missing data for that individual parameter. fSCIG, facilitated subcutaneous immunoglobulin (PNG 79 kb)

High reoslution image (EPS 78 KB)

Supplemental Fig. S2 fSCIG infusion interval over 36 months of follow-up, excluding patients in the ramp-up phase at the inclusion visit. NA, not applicable as the patients received only 1 fSCIG infusion to date. fSCIG, facilitated subcutaneous immunoglobulin (PNG 147 kb)

High reoslution image (EPS 143 KB)

Supplemental Fig. S3 fSCIG infusion interval over 36 months of follow-up, excluding patients in the ramp-up phase at the inclusion visit. NA, not applicable as the patients received only 1 fSCIG infusion to date. fSCIG, facilitated subcutaneous immunoglobulin (PNG 125 kb)

High resolution image (EPS 122 KB)

Supplementary file4 (DOCX 17 KB)

Supplementary file5 (DOCX 17 KB)

Supplementary file6 (DOCX 15 KB)

Supplementary file7 (DOCX 15 KB)

Supplementary file8 (DOCX 20 KB)

## Data Availability

The data that support the findings of this study are available on request from the corresponding author [DP]. Data collection was prospective (using retrospective documentation of anamnestic information) using only available information based on patient charts, diaries, or patient interviews (as available).
