# Supplementary figures and images for: Facilitated Subcutaneous Immunoglobulin Treatment in Patients with Immunodeficiencies: the FIGARO Study
Source: J Clin Immunol. 2023 Apr 10;43(6):1259–71. doi: 10.1007/s10875-023-01470-2 (PMC10088636; doi:10.1007/s10875-023-01470-2)

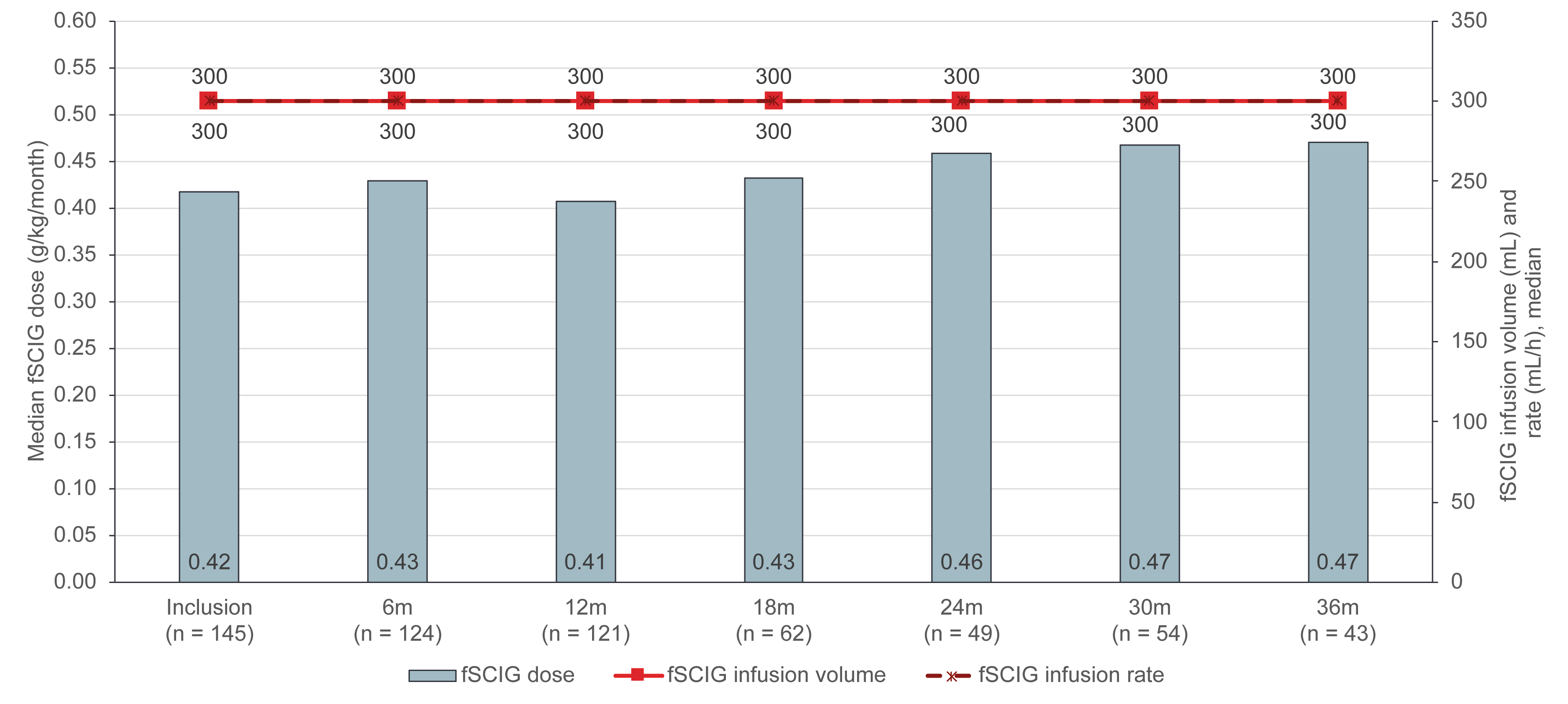

Supplement: Supplementary file 1 — fSCIG dose, infusion volume, and rate over 36 months of follow-up, excluding patients in the ramp-up phase at the inclusion visit. n values represent number of patients at each visit; n values for each parameter may differ slightly due to missing data for that individual parameter. fSCIG, facilitated subcutaneous immunoglobulin (PNG 79 kb) [file 10875_2023_1470_Fig6_ESM.png]

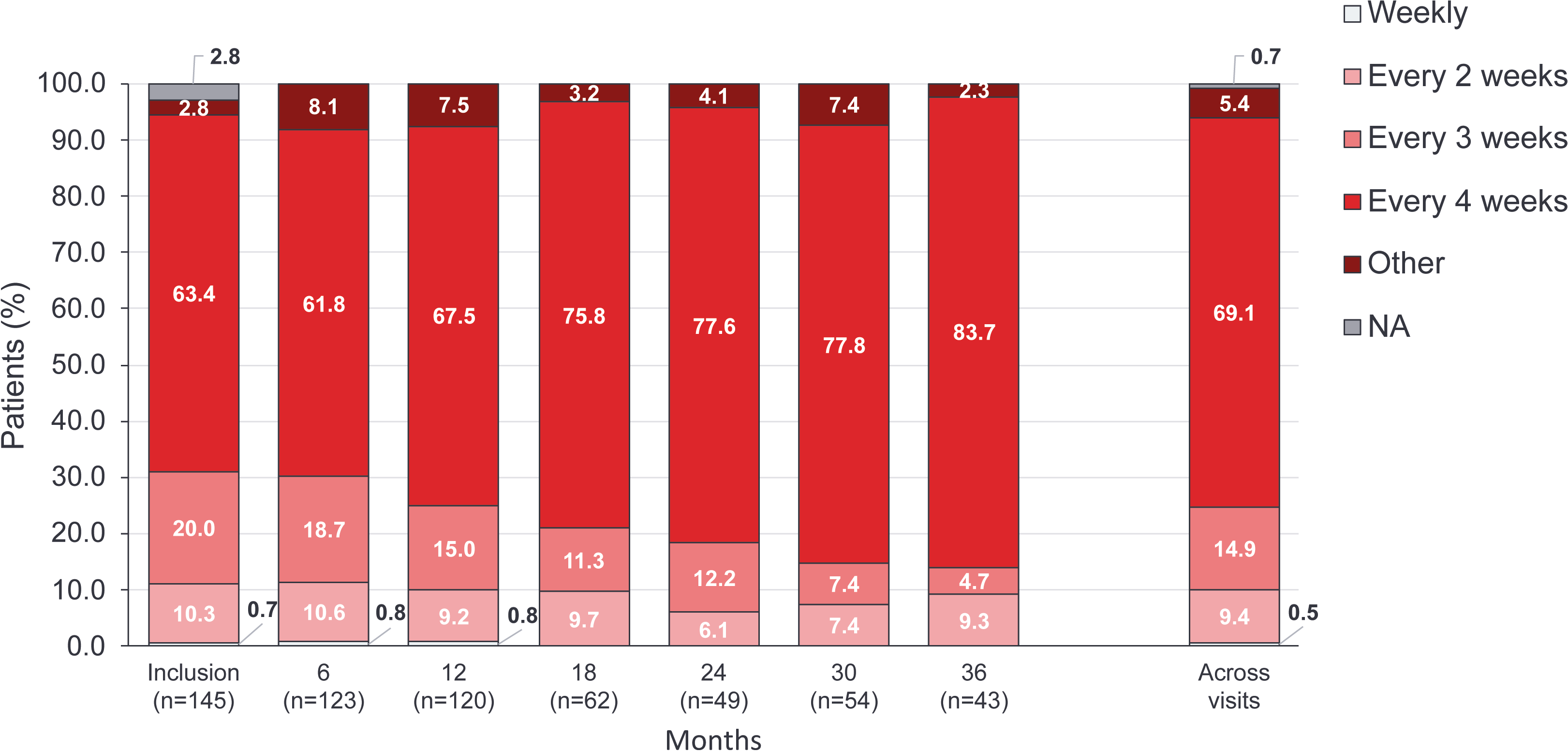

Supplement: Supplementary file 3 — fSCIG infusion interval over 36 months of follow-up, excluding patients in the ramp-up phase at the inclusion visit. NA, not applicable as the patients received only 1 fSCIG infusion to date. fSCIG, facilitated subcutaneous immunoglobulin (PNG 147 kb) [file 10875_2023_1470_Fig7_ESM.png]

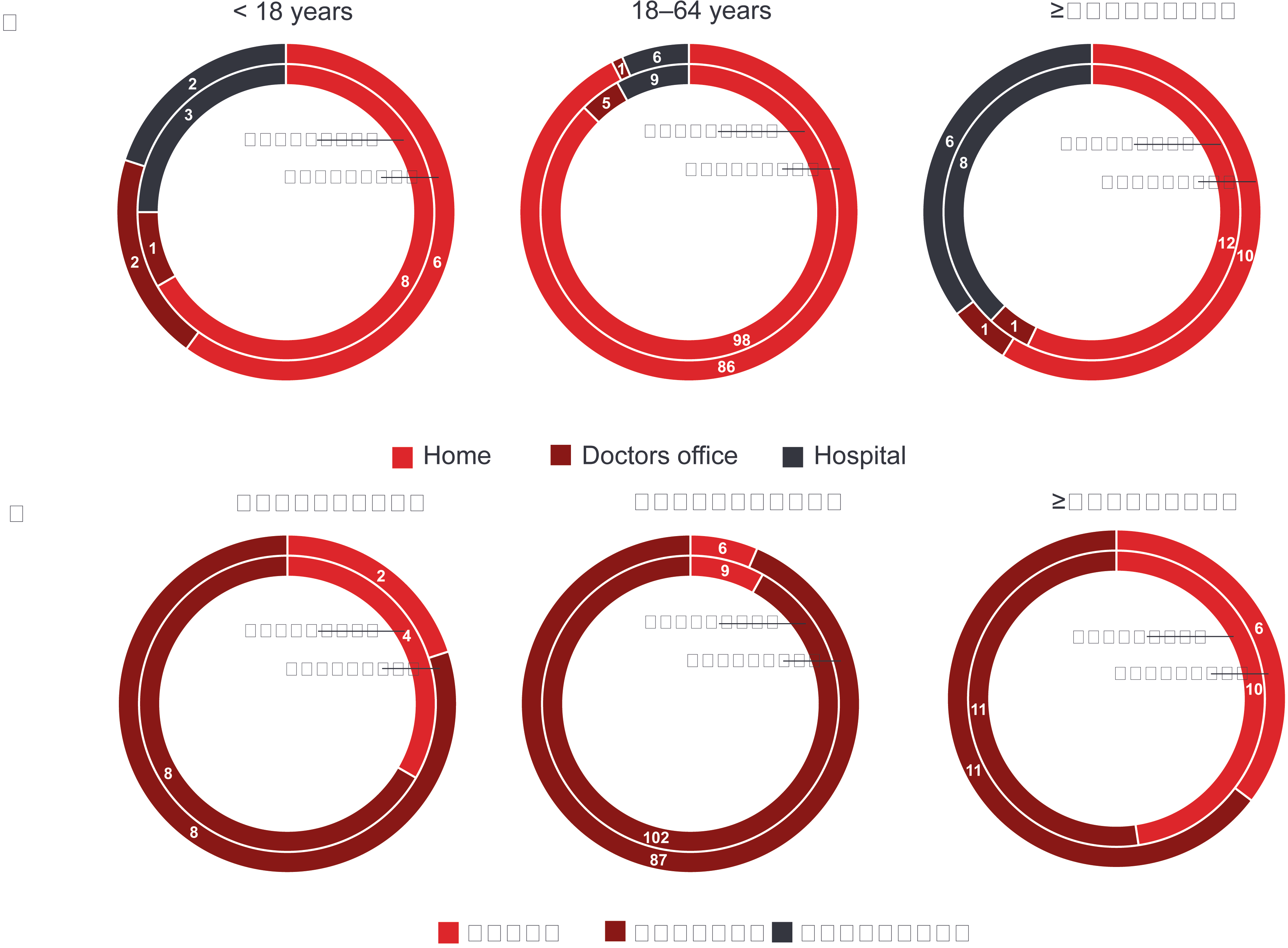

Supplement: Supplementary file 5 — fSCIG infusion interval over 36 months of follow-up, excluding patients in the ramp-up phase at the inclusion visit. NA, not applicable as the patients received only 1 fSCIG infusion to date. fSCIG, facilitated subcutaneous immunoglobulin (PNG 125 kb) [file 10875_2023_1470_Fig8_ESM.png]
