# Supplementary material for: Facilitated Subcutaneous Immunoglobulin Treatment in Patients with Immunodeficiencies: the FIGARO Study
Source: J Clin Immunol. 2023 Apr 10;43(6):1259–71. doi: 10.1007/s10875-023-01470-2 (PMC10088636; doi:10.1007/s10875-023-01470-2)
Supplement: Supplementary file 7 — Supplementary file4 (DOCX 17 KB) [file 10875_2023_1470_MOESM4_ESM.docx]

**Title:** Facilitated Subcutaneous Immunoglobulin Treatment in Patients with Immunodeficiencies: the FIGARO Study

**Journal:** Journal of Clinical Immunology

**Authors:** Michael Borte, Leif G. Hanitsch, Nizar Mahlaoui, Maria Fasshauer, Dörte Huscher, Matthaios Speletas, Maria Dimou, Marta Kamieniak, Corinna Hermann, David Pittrow, Cinzia Milito

**Corresponding author:**

David Pittrow

Institute for Clinical Pharmacology, Medical Faculty,

Technical University of Dresden, Dresden, Germany

[david.pittrow@mailbox.tu-dresden.de](mailto:david.pittrow@mailbox.tu-dresden.de)

**Supplemental Table 1. Concomitant disease state at inclusion by indication (MeDRA code reported in ≥2 patients)**

| **Concomitant diseases, n (%)** | **PID** | **SID** | **Total** |
| --- | --- | --- | --- |
| Adipositas | 3 (1.6) | 0 (0) | 3 (1.3) |
| Anemia | 3 (1.6) | 1 (2.9) | 4 (1.8) |
| Chronic bronchitis | 2 (1.0) | 1 (2.9) | 3 (1.3) |
| Chronic rhinitis | 2 (1.0) | 0 (0) | 2 (0.9) |
| Chronic sinusitis | 10 (5.2) | 2 (5.9) | 12 (5.3) |
| Cholecystolithiasis | 1 (0.5) | 1 (2.9) | 2 (0.9) |
| COPD | 2 (1.0) | 0 (0) | 2 (0.9) |
| Dietary B12 deficiency | 2 (1.0) | 0 (0) | 2 (0.9) |
| Dyslipidemia | 2 (1.0) | 2 (5.9) | 4 (1.8) |
| Endometriosis | 1 (0.5) | 1 (2.9) | 2 (0.9) |
| Gastroesophageal reflux disease | 2 (1.0) | 0 (0) | 2 (0.9) |
| Goiter nodular | 2 (1.0) | 0 (0) | 2 (0.9) |
| Hashimoto’s disease | 2 (1.0) | 0 (0) | 2 (0.9) |
| Herniated disk NOS | 3 (1.6) | 0 (0) | 3 (1.3) |
| Hypercholesterolemia | 2 (1.0) | 1 (2.9) | 3 (1.3) |
| Hyperuricemia | 2 (1.0) | 0 (0) | 2 (0.9) |
| Iron deficiency | 2 (1.0) | 0 (0) | 2 (0.9) |
| ITP | 2 (1.0) | 0 (0) | 2 (0.9) |
| Leukopenia | 2 (1.0) | 1 (2.9) | 3 (1.3) |
| Lymphadenopathy | 4 (2.1) | 1 (2.9) | 5 (2.2) |
| Lymphopenia | 1 (0.5) | 1 (2.9) | 2 (0.9) |
| Megaloblastic anemia | 2 (1.0) | 0 (0) | 2 (0.9) |
| Nasal polyps | 3 (1.6) | 0 (0) | 3 (1.3) |
| Obesity | 2 (1.0) | 0 (0) | 2 (0.9) |
| Osteopenia | 2 (1.0) | 0 (0) | 2 (0.9) |
| Pansinusitis | 8 (4.2) | 0 (0) | 8 (3.6) |
| Pernicious anemia | 3 (1.6) | 0 (0) | 3 (1.3) |
| Primary biliary cirrhosis | 1 (0.5) | 1 (2.9) | 2 (0.9) |
| Psoriasis | 2 (1.0) | 0 (0) | 2 (0.9) |
| Splenomegaly | 11 (5.8) | 3 (8.8) | 14 (6.2) |
| Thrombocytopenia | 1 (0.5) | 1 (2.9) | 2 (0.9) |
| Vitamin B12 deficiency | 2 (1.0) | 0 (0) | 2 (0.9) |
| Vitiligo | 3 (1.6) | 0(0) | 3 (1.3) |

Multiple responses possible. COPD, chronic obstructive pulmonary disease; ITP, immune thrombocytopenia; NOS, not otherwise specified; PID, primary immunodeficiency disease; SID, secondary immunodeficiency disease.
