# Supplementary material for: Facilitated Subcutaneous Immunoglobulin Treatment in Patients with Immunodeficiencies: the FIGARO Study
Source: J Clin Immunol. 2023 Apr 10;43(6):1259–71. doi: 10.1007/s10875-023-01470-2 (PMC10088636; doi:10.1007/s10875-023-01470-2)
Supplement: Supplementary file 8 — Supplementary file5 (DOCX 17 KB) [file 10875_2023_1470_MOESM5_ESM.docx]

**Title:** Facilitated Subcutaneous Immunoglobulin Treatment in Patients with Immunodeficiencies: the FIGARO Study

**Journal:** Journal of Clinical Immunology

**Authors:** Michael Borte, Leif G. Hanitsch, Nizar Mahlaoui, Maria Fasshauer, Dörte Huscher, Matthaios Speletas, Maria Dimou, Marta Kamieniak, Corinna Hermann, David Pittrow, Cinzia Milito

**Corresponding author:**

David Pittrow

Institute for Clinical Pharmacology, Medical Faculty,

Technical University of Dresden, Dresden, Germany

[david.pittrow@mailbox.tu-dresden.de](mailto:david.pittrow@mailbox.tu-dresden.de)

**Supplemental Table 2. fSCIG dosing and infusion parameters at inclusion and 12 months by age subgroups, excluding patients in the ramp-up phase at the inclusion visit**

| Parameter, median (range) | **<18 years** | | **18–64 years** | | **≥65 years** | | **Total** | |
| --- | --- | --- | --- | --- | --- | --- | --- | --- |
|  | **Inclusion** | **12 months** | **Inclusion** | **12 months** | **Inclusion** | **12 months** | **Inclusion** | **12 months** |
|  | (n=12) | (n=10) | (n=112) | (n=94) | (n=21) | (n=17) | (n=145) | (n=121) |
| Total fSCIG dose at the most recent infusion, g | 13.8  (10.0–30.0) | 17.5  (10.0–40.0) | 30.0  (10.0–60.0) | 30.0 (10.0–60.0) | 30.0 (20.0–50.0) | 30.0 (15.0–50.0) | 30.0 (10.0–60.0) | 30.0 (10.0–60.0) |
| fSCIG total monthly dose, g | 15.0 (10.0–30.0) | 20.0 (10.0–40.0) | 30.0 (10.0–75.0) | 30.0 (10.0–60.0) | 30.0 (20.0–50.0) | 30.0 (1.0–50.0) | 30.0 (10.0–75.0) | 30.0 (1.0–60.0) |
| fSCIG dose, g/kg/month | 0.500 (0.106–0.833) | 0.500 (0.341–0.645) | 0.412 (0.169–0.816) | 0.421 (0.116–0.857) | 0.410 (0.250–0.545) | 0.349 (0.012–0.526) | 0.417 (0.106–0.833) | 0.407 (0.012–0.857) |
| Total fSCIG infusion volume^a^, mL | 137.5 (100.0–300.0) | 175.0 (100.0–400.0) | 300.0 (10.0–600.0) | 300.0 (25.0–600.0) | 300.0 (40.0–350.0) | 300.0 (30.0–400.0) | 300.0 (10.0–600.0) | 300.0 (25.0–600.0) |
| fSCIG maximum infusion rate, mL/h | 126.5 (10.0–300.0) | 171.0 (86.0–300.0) | 300.0 (60.0–300.0) | 300.0 (60.0–320.0) | 300.0 (240.0–300.0) | 275.0 (240.0–300.0) | 300.0 (10.0–300.0) | 300.0 (60.0–320.0) |
| Infusion interval, n (%)  Weekly  Every 2 weeks  Every 3 weeks  Every 4 weeks  Other  NA^b^ | 0  0  3 (25.0)  9 (75.0)  0  0 | 0  0  0  10 (100.0)  0  0 | 1 (0.9)  15 (13.4)  25 (22.3)  66 (58.9)  2 (1.8)  3 (2.7) | 1 (1.1)  11 (11.8)  17 (18.3)  58 (62.4)  6 (6.5)  0 | 0  0  1 (4.8)  17 (81.0)  2 (9.5)  1 (4.8) | 0  0  1 (5.9)  13 (76.5)  3 (17.6)  0 | 1 (0.7)  15 (10.3)  29 (20.0)  92 (63.4)  4 (2.8)  4 (2.8) | 1 (0.8)  11 (9.2)  18 (15.0)  81 (67.5)  9 (7.5)  0 |

^a^Total infusion volume over all sites per patient. ^b^ NA, not applicable as the patient received only one fSCIG infusion to date.
fSCIG, facilitated subcutaneous immunoglobulin.
