# Supplementary material for: Facilitated Subcutaneous Immunoglobulin Treatment in Patients with Immunodeficiencies: the FIGARO Study
Source: J Clin Immunol. 2023 Apr 10;43(6):1259–71. doi: 10.1007/s10875-023-01470-2 (PMC10088636; doi:10.1007/s10875-023-01470-2)
Supplement: Supplementary file 9 — Supplementary file6 (DOCX 15 KB) [file 10875_2023_1470_MOESM6_ESM.docx]

**Title:** Facilitated Subcutaneous Immunoglobulin Treatment in Patients with Immunodeficiencies: the FIGARO Study

**Journal:** Journal of Clinical Immunology

**Authors:** Michael Borte, Leif G. Hanitsch, Nizar Mahlaoui, Maria Fasshauer, Dörte Huscher, Matthaios Speletas, Maria Dimou, Marta Kamieniak, Corinna Hermann, David Pittrow, Cinzia Milito

**Corresponding author:**

David Pittrow

Institute for Clinical Pharmacology, Medical Faculty,

Technical University of Dresden, Dresden, Germany

[david.pittrow@mailbox.tu-dresden.de](mailto:david.pittrow@mailbox.tu-dresden.de)

**Supplemental Table 3**. **Local and systemic adverse reactions by age subgroup**

| n (%) | **< 18 years** | | **18–64 years** | | **≥65 years** | | **Total** | |
| --- | --- | --- | --- | --- | --- | --- | --- | --- |
|  | **Inclusion** | **12 months** | **Inclusion** | **12 months** | **Inclusion** | **12 months** | **Inclusion** | **12 months** |
|  | (n=15) | (n=12) | (n=120) | (n=99) | (n=21) | (n=17) | (n=156) | (n=128) |
| ADR associated with fSCIG infusion | 2 (13.3) | 1 (8.3) | 27 (22.5) | 19 (19.4) | 1 (4.8) | 1 (5.9) | 30 (19.2) | 21 (16.5) |
| ADR local^a^ | 2 (13.3) | 1 (8.3) | 22 (18.3) | 14 (14.1) | 1 (4.8) | 0 | 25 (16.0) | 15 (11.7) |
| ADR systemic^b^ | 0 | 0 | 12 (10.0) | 10 (10.1) | 1 (4.8) | 1 (5.9) | 13 (8.3) | 11 (8.6) |

Multiple reactions possible. ^a^Local (infusion site) includes infusion site erythema, inflammation, infusion site itching. ^b^Systemic (generalized or non-infusion site) includes acute diarrhea, aseptic meningitis, chills, dizziness, drowsiness, fatigue, fever, fever chills, flu-like symptoms, headache, hypertension, itching, malaise, vasovagal reaction, weakness.
ADR, adverse drug reactions; fSCIG, facilitated subcutaneous immunoglobulin.
