# Supplementary material for: Facilitated Subcutaneous Immunoglobulin Treatment in Patients with Immunodeficiencies: the FIGARO Study
Source: J Clin Immunol. 2023 Apr 10;43(6):1259–71. doi: 10.1007/s10875-023-01470-2 (PMC10088636; doi:10.1007/s10875-023-01470-2)
Supplement: Supplementary file 10 — Supplementary file7 (DOCX 15 KB) [file 10875_2023_1470_MOESM7_ESM.docx]

**Title:** Facilitated Subcutaneous Immunoglobulin Treatment in Patients with Immunodeficiencies: the FIGARO Study

**Journal:** Journal of Clinical Immunology

**Authors:** Michael Borte, Leif G. Hanitsch, Nizar Mahlaoui, Maria Fasshauer, Dörte Huscher, Matthaios Speletas, Maria Dimou, Marta Kamieniak, Corinna Hermann, David Pittrow, Cinzia Milito

**Corresponding author:**

David Pittrow

Institute for Clinical Pharmacology, Medical Faculty,

Technical University of Dresden, Dresden, Germany

[david.pittrow@mailbox.tu-dresden.de](mailto:david.pittrow@mailbox.tu-dresden.de)

**Supplemental Table 4. Adverse reactions by administration setting, by age subgroup**

| ADR associated with fSCIG infusion, n (%) | **< 18 years** | | **18–64 years** | | **≥65 years** | | **Total** | |
| --- | --- | --- | --- | --- | --- | --- | --- | --- |
|  | **Inclusion** | **12 months** | **Inclusion** | **12 months** | **Inclusion** | **12 months** | **Inclusion** | **12 months** |
|  | (n=15) | (n=12) | (n=120) | (n=99) | (n=21) | (n=17) | (n=156) | (n=128) |
| Patient’s home | 1 (12.5) | 1 (12.5) | 22 (22.4) | 19 (20.9) | 0 | 0 | 23 (19.5) | 20 (18.3) |
| Doctor’s office/hospital | 1 (14.3) | 0 | 5 (22.7) | 0 | 1 (11.1) | 1 (14.3) | 7 (18.4) | 1 (5.6) |

Multiple reactions possible.
ADR, adverse drug reactions; fSCIG, facilitated subcutaneous immunoglobulin.
