# Supplementary material for: Facilitated Subcutaneous Immunoglobulin Treatment in Patients with Immunodeficiencies: the FIGARO Study
Source: J Clin Immunol. 2023 Apr 10;43(6):1259–71. doi: 10.1007/s10875-023-01470-2 (PMC10088636; doi:10.1007/s10875-023-01470-2)
Supplement: Supplementary file 11 — Supplementary file8 (DOCX 20 KB) [file 10875_2023_1470_MOESM8_ESM.docx]

**Title:** Facilitated Subcutaneous Immunoglobulin Treatment in Patients with Immunodeficiencies: the FIGARO Study

**Journal:** Journal of Clinical Immunology

**Authors:** Michael Borte, Leif G. Hanitsch, Nizar Mahlaoui, Maria Fasshauer, Dörte Huscher, Matthaios Speletas, Maria Dimou, Marta Kamieniak, Corinna Hermann, David Pittrow, Cinzia Milito

**Corresponding author:**

David Pittrow

Institute for Clinical Pharmacology, Medical Faculty,

Technical University of Dresden, Dresden, Germany

[david.pittrow@mailbox.tu-dresden.de](mailto:david.pittrow@mailbox.tu-dresden.de)

**Supplemental Table 5. ASBI events in the 12 months prior to inclusion and during follow-up**

| **Number of events, n (%)** | **12 months prior to inclusion** | | | **Follow-up^a^** | | | | |
| --- | --- | --- | --- | --- | --- | --- | --- | --- |
|  |  |  |  | **6 months** | **12 months** | **18 months** | **24 months** | **30 months** |
|  | **PID** | **SID** | **Total** | **PID** | **PID** | **PID** | **PID** | **PID** |
| Acute bronchitis | - | - | - | - | 2 (40.0) | - | 2 (25.0) | - |
| Acute diarrhea | - | - | - | - | - | 1 (20.0) | 1 (12.5) | - |
| Bronchitis | - | - | - | - | - | 2 (40.0) | - | - |
| Diarrhea, *Clostridium difficile* | - | 1 (7.1) | 1 (3.8) | - | - | - | - | - |
| Febrile neutropenia | - | 2 (14.3) | 2 (7.7) | - | - | - | - | - |
| Flu-like symptoms | - | - | - | 1 (14.3) | - | - | 1 (12.5) | - |
| Increased susceptibility to infections NOS | - | 1 (7.1) | 1 (3.8) | - | - | - | - | - |
| Infection of kidney, unspecified | - | 1 (7.1) | 1 (3.8) | - | - | - | - | - |
| Lower respiratory tract infection | - |  | - | - | - | - | 1 (12.5) | 2 (66.7) |
| Lower urinary tract infection | - | - | - | - | - | - | 1 (12.5) |  |
| Lung abscess | - | - | - | 1 (14.3) | - | - | - | - |
| Pneumonia | 10 (83.3) | 5 (35.7) | 15 (57.7) | 1 (14.3) | 3 (60.0) | 1 (20.0) | - | - |
| Pseudomonas infection NOS | - | 1 (7.1) | 1 (3.8) | - | - | - | - | - |
| Sinusitis | - | - | - | 3 (42.9) | - | - | - | - |
| Tonsillitis | - | 2 (14.3) | 2 (7.7) |  | - | - | - | - |
| Upper respiratory tract infection | - | - | - | 1 (14.3) | - | - | 2 (25.0) | - |
| Urinary tract infection | - | - | - | - | - | - | - | 1 (33.3) |
| Puncture site abscess | - | 1 (7.1) | 1 (3.8) | - | - | - | - | - |
| Enterococcal sepsis | 1 (8.3) | - | 1 (3.8) | - | - | - | - | - |
| Pleuropneumonia | 1 (8.3) | - | 1 (3.8) | - | - | - | - | - |
| Acute bacterial exacerbation of chronic sinusitis | - | - | - | - | - | 1 (20.0) | - | - |
| **Total** | **12 (100)** | **14 (100)** | **26 (100)** | **7 (100)** | **5 (100)** | **5 (100)** | **8 (100)** | **3 (100)** |

Multiple responses possible.

^a^ASBI events occurred only in the PID cohort during follow-up.

ASBI, acute severe bacterial infection; NOS, not otherwise specified
